# Effectiveness of a *Staphylococcus aureus* mupirocin decolonization protocol in a neonatal intensive care unit

**DOI:** 10.1017/ash.2023.220

**Published:** 2023-09-29

**Authors:** Andrea Ankrum, Felicia Scaggs Huang, Joshua Schaffzin

## Abstract

**Background:** *Staphylococcus aureus* infections are a significant cause of morbidity in neonatal intensive care units (NICUs), and they are typically preceded by nasal colonization. Mupirocin decolonization protocols as an infection prevention tool can halt *S. aureus* outbreaks and prevent infections. We examined the effect of a mupirocin treatment protocol on *S. aureus* incidence, prevalence, decolonization, and infections in a level IV NICU. **Methods:** We conducted a retrospective before-and-after observational study from June 1, 2018, to May 31, 2020. Beginning June 1, 2019, patients identified with either methicillin-sensitive or -resistant *S. aureus* (MSSA or MRSA) received mupirocin ointment to bilateral nares 3 times daily for 5 days. Patients with central lines or surgical incisions were treated every 30 days. All NICU patients were screened weekly unless positive for MRSA. We defined MSSA decolonization as 3 consecutive negative screens following mupirocin treatment. We calculated monthly *S. aureus* incidence as any new positive per 1,000 patient days and monthly prevalence as a percentage of the average daily number of patients with *S. aureus* per the average monthly census. Total number of infections were compared. Statistical significance was determined using a 2 sample proportions test and *P* < .05. Decolonization was calculated as percentage of occurrences among treated patients. **Results:** Overall, 190 patients received mupirocin; 142 patients received 1 course and 48 received 2 or more courses. There was no difference in incidence of MSSA (*P* = .09), but prevalence decreased from 18.8% to 14.4% (*P* < .05) (Fig. 1). Of 66 patients with MSSA, 35% were decolonized. An additional mupirocin treatment for 16 of these patients had a 50% decolonization rate. For MRSA, incidence decreased from 0.24 to 0.20 (*P* = .36) and prevalence decreased from 21.9% to 18.6% (*P* < .05) (Fig. 1). There was no statistical difference in the number of total *S. aureus* infections (*P* = .91) or when stratified by MSSA (*P* = .72) or MRSA (*P* = .82). In the postmupirocin population, there were 5 MRSA and 7 MSSA infections. Of the MSSA-infected patients, 6 remained colonized at the time of infection. **Conclusions:** Implementing a single mupirocin treatment course for *S. aureus* decolonization in NICU was only effective in one-third of patients and had no effect on infection occurrence. Changes in incidence and prevalence could be confounded by other infection prevention practices. Further study is needed to determine whether continued screening and additional mupirocin treatment could improve effectiveness of *S. aureus* decolonization programs.

**Figure:**
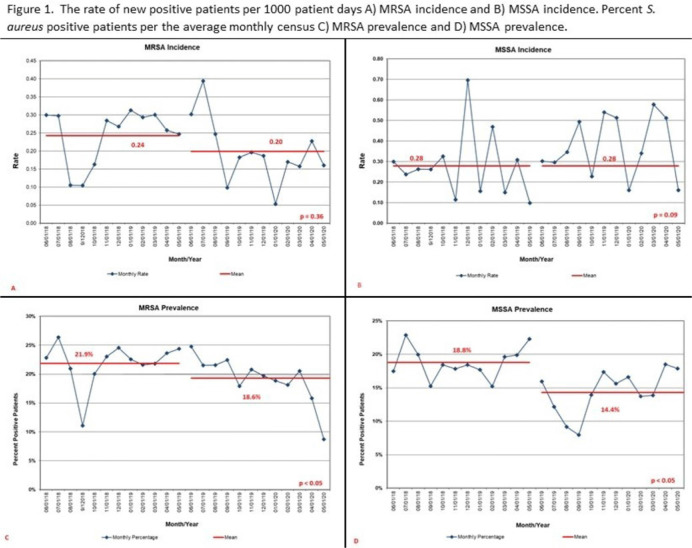

**Disclosure:** None

